# The putative effects of D-Aspartic acid on blood testosterone levels: A systematic review

**Published:** 2017-01

**Authors:** Farzad Roshanzamir, Seyyed Morteza Safavi

**Affiliations:** 1 *Noncommunicable Diseases Research Center, Fasa University of Medical Sciences, Fasa, Iran.*; 2 *Department of Clinical Nutrition, School of Nutrition and Food Sciences, Isfahan University of Medical Sciences, Isfahan, Iran*

**Keywords:** D-Aspartic acid, Testosterone, Systematic review

## Abstract

**Background:**

D-Aspartic acid (D-Asp) is in invertebrate and vertebrate neuroendocrine tissues, where it carries out important physiological functions. Recently, it has been reported that D-Asp is involved in the synthesis and release of testosterone and is assumed can be used as a testosterone booster for infertile men, and by athletes to increase muscle mass and strength.

**Objective:**

The aim of this review is to summarize available evidence related to the effects of D-Asp on serum testosterone levels.

**Materials and Methods:**

We conducted a systematic review of all type studies, which evaluated the effect of the D-Asp on blood testosterone including published papers until October 2015, using PubMed, ISI Web of Science, ProQuest and Scopus database.

**Results:**

With 396 retrieved records, 23 animal studies and 4 human studies were included. In vivo and in vitro animal studies revealed the effect of D-Asp depending on species, sex and organ-specific. Our results showed that exogenous D-Asp enhances testosterone levels in male animal’s studies, whereas studies in human yielded inconsistent results. The evidence for this association in man is still sparse, mostly because of limited number and poor quality studies.

**Conclusion:**

There is an urgent need for more and well-designed human clinical trials with larger sample sizes and longer duration to investigate putative effects of D-Asp on testosterone concentrations.

## Introduction

D-Aspartic acid (D-Asp) is an endogenous amino acid occurring in several tissues and cells of both invertebrates and vertebrates (1). D-Asp was first detected in the brain and optic lobes of the cephalopod mollusk Octopus vulgaris and later in the nervous and endocrine systems of various animal phyla such as crustaceans, amphibians, reptiles, fish, chicken, rat, and man (2-13). 

In rat brain, this amino acid has been localized in various neurons, including the frontal cortex and hippocampus (14, 15). In human, D-Asp has been found in both fetal and adult brains as well as in the cerebrospinal fluid of adult individuals (12). Within the nervous system this amino acid is concentrated in the axon terminal and in synaptic vesicles suggesting it has a role in neurotransmission and neurosecretory activities (16, 17). In addition, D-Asp levels increase transiently during the last stage of embryonic life and decrease rapidly after delivery in several regions of human or rat brain (11). These data support that D-Asp may participate in during the development and neurogenesis of embryonic brain (18). 

D-Asp also occurs in endocrine systems particularly in those associated with the reproductive system such as adrenal gland, pineal gland, pituitary gland, and testis, where it increases during postnatal and adult life (1, 19-22). D-Asp plays an important role in the biosynthesis and/or secretion of hormones in endocrine glands. D-Asp induces prolactin release in the anterior pituitary gland, modulates oxytocin and vasopressin production in posterior pituitary gland, suppresses melatonin secretion in the pineal gland, modulates steroidogenesis in the adrenal gland, and regulates testosterone production (23-25). These functions reflect the D-Asp involvement in the neuroendocrine system (26). 

D-Asp is synthesized endogenously where it is needed from L-Asp by an aspartate racemase (27). In amphibian testis, the changes of this enzyme are significant, and racemase activity peaks in the reproductive period when D-Asp peaks testosterone levels are the highest during spermatogenesis and reproduction. Thus, D-Asp contributes to the production of more testosterone in the testis (28). Several studies have reported that D-Asp regulates the release and synthesis of testosterone through multiple pathways of the hypothalamic-pituitary-gonadal (HPG) axis (29). 

D-aspartate methyltransferase (NMDA synthetase) converts D-Asp to N-methyl D-aspartic acid (NMDA) which is directly responsible for hormone release. In adult male rats, NMDA has been shown to act on the hypothalamus, inducing an increase of gonadotropin releasing hormone (GnRH); in the pituitary gland, GnRH induces LH release; and in the gonads, LH facilitates testosterone release (30). D-Asp could directly up-regulate testosterone production in rat testis by increasing cAMP levels and activate steroidogenic acute regulatory protein (StAR) and gene expressions (31). StAR protein involves in cholesterol translocation to the inner mitochondrial membrane and is a key regulatory protein playing an essential role in steroidogenesis (32). 

D-Asp acid is currently recommended as a viable product to significantly raise testosterone although this recommendation appears not to be based on systematic reviews of the evidence. Accordingly, we carried out a systematic review of the scientific evidence to determine the effect of D-Asp on testosterone levels and to provide a comprehensive summary of the molecular and biochemical mechanisms underlying the relationship between D-Asp and testosterone levels.

## Materials and methods

We included all types of studies describing any association between D-Asp and testosterone until October 2015. The search strategy for PubMed and Web of Science was no limit and for ProQuest and Scopus with selection “Anywhere expect full text” and “Article Title, Abstract, Keywords” was set, respectively (Table I). Additional articles were identified from reference lists of included studies and relevant reviews. 

## Results

**Figure 1:**
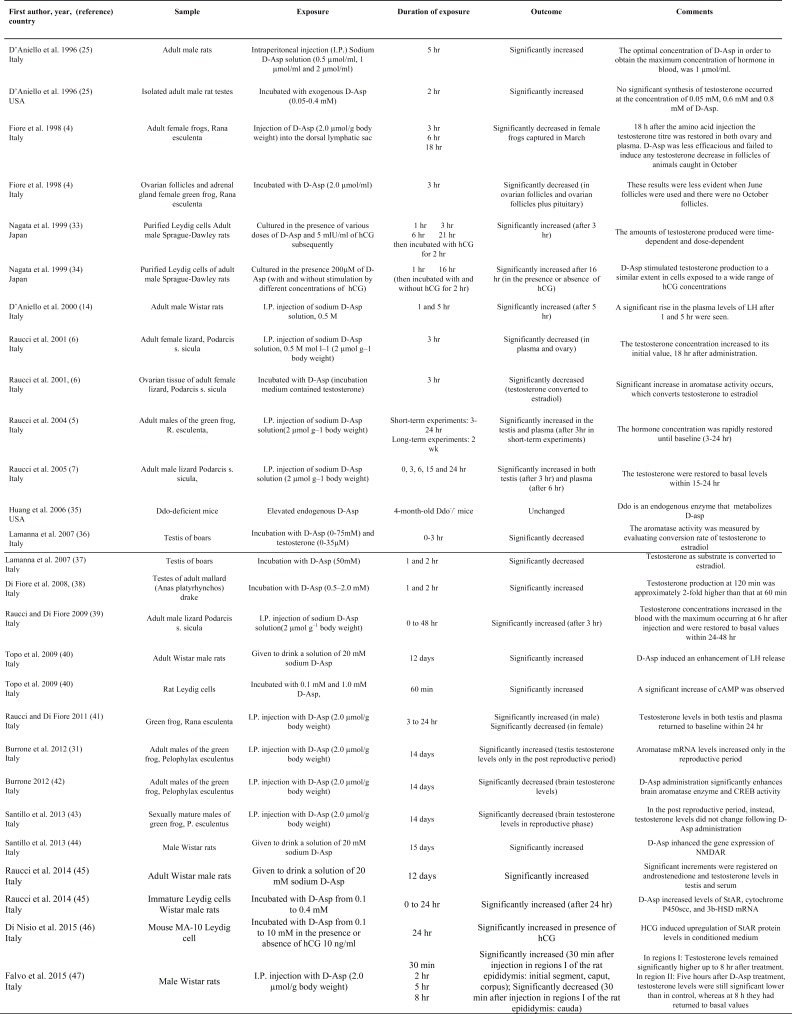
The flow diagram of study selection process.

The flow diagram of the studies throughout the selection process is shown in Figure 1. We retrieved 96 references from the PubMed database, 127 from Web of Science databases, 76 from the ProQuest and 97 from the Scopus database. The title and abstract of various articles were initially screened and evaluated and only 96 articles were included as potentially relevant. An examination of the full text of these articles resulted in the exclusion of 25 studies. After hand searching of the bibliographies of these articles and relevant reviews, we identified 2 additional articles, leaving 27 articles for final inclusion: 4 in vivo human studies, and 23 animal studies. 

The characteristics and outcomes of the included animal and human studies are presented in table II, III respectively, and they are categorized by the first author’s name, study type and duration of the follow-up period and sorted by the year of publication. Quantitative meta-analysis was not done because of various designs of included studies and small amount of human studies. 

**In vivo and in vitro animal studies**

In vivo animal studies were carried out on male rats, male green frogs, male lizards, and male mallards (4, 5, 7, 14, 25, 31, 38-44). These studies showed that D-Asp increases testosterone levels in the blood. These results are contrary from those were carried out on females green frog and female lizards, in which these differences assume different effect of D-Asp depending on species, sex and organ-specific (4, 6, 41). In addition, in a study by Huang *et al*. serum testosterone concentrations not altered in D-Asp oxidase (Ddo) knockout mice. Ddo is an endogenous enzyme and is responsible for the metabolizing of D-Asp (35).

In vitro studies clarified the functions of D-Asp in steroidogenesis. Nagata *et al* demonstrated that D-Asp increases human chorionic gonadotropin (hCG)-stimulated testosterone synthesis in purified rat Leydig cells. hCG via activation of adenylate cyclase activates the cAMP translocation of cholesterol to the inner mitochondrial membrane which is required for testosterone synthesis (33). Also they have shown that D-Asp and hCG synergistically up-regulate the production of testosterone in rat Leydig cells, apparently by increasing gene expression StAR in purified rat Leydig cells (31, 34, 45, 46). 

D’Aniello *et al* demonstrated that D-Asp can cause synthesis and release of GnRH from hypothalamus in two ways: direct inducing action of the hypothalamus and converting to NMDA (14). Other studies showed that D-Asp directly inhances aromatase activity (5, 6, 31, 36, 42, 43, 47).

**Human studies**

Topo *et al* carried out a clinical trial on healthy male volunteers aged between 27 and 37 years at the IVF (in vitro fertilization) unit (40). In the intervention group, 23 participants consumed 3.12 gr of sodium D-Asp for 12 consecutive days. The LH and testosterone levels after 6 days of treatment did not significantly increase, after 12 days of treatment significantly increased, by 33% and 42% respectively and three days after sodium D-Asp suspension increased higher than that of basal levels. Topo *et al* suggested that consumed D-Asp may also be remained in the testis, and it continued to stimulate the testosterone production in the testis. In this study the mean baseline testosterone within 25% of the lower clinical range (3-10 ng/mL), and D-Asp supplementation elevated testosterone levels to approximately 50% of the clinical range (4.5-6.4 ng/mL) (52). 

Inclusion and exclusion criteria were not specified and it is a methodological limitation for this study. In addition, except the age, no details of the participants were reported (e.g. weight, physical activity). Significant differences between two groups have not been analyzed. It seems the allocation of participants to the study groups was not random. Thus, the two groups may differed in various baseline characteristics, such as diet, drugs or other supplement consumption, weight, physical activity. These baseline variables were not controlled. No adjustment for potential confounders was performed. No adverse events were reported.

In a randomized, double-blind interventional study by Willoughby *et al*, twenty apparent healthy and heavy resistance-trained men were enrolled (49). The participants trained 4 times/week while orally ingesting either 3 gr of placebo or D-Asp in the morning upon waking. After 28 days D-Asp supplementation had no effect on muscle strength, body mass and serum hormones (total testosterone, free testosterone, LH, GnRH and estradiol). Unlike the previous study, participant demographics had been well described such as age, weight and height. All participants passed a mandatory medical screening. 

Exclusion criteria were consuming any nutritional supplements (excluding multivitamins) three months before the study. Adverse events were reported. three participants reported feelings of irritability, nervousness, rapid heart rate, and headache. There were no significant differences between groups for total calories or for the intake of protein, carbohydrate, and fat. The limitations of this study are the small sample size and the reliance on self-report for dietary intake and supplement compliance.

Contrary to the study of Topo *et al* in this study D-Asp supplementation had no effects on serum testosterone (40). This discrepancy may be due to the difference in baseline levels of total testosterone that in this study were close to the maximum clinical range (approximately 8 ng/mL, which is within 25% of the upper clinical range) and in the study of Topo *et al* were close to the minimum clinical range (approximately 4.5 ng/mL, which is within 25% of the lower clinical range) (40). Thus, D-Asp supplementation may only be effective with lower testosterone levels.

Another clinical trial study, by Bloomer *et al* investigated the effect of DAA/sodium nitrate/ vitamin D3 supplement on bBlood total and free testosterone and nitrate/nitrite before and after 14 and 28 days in 10 overweight or obese men with the average of 42 yr (50). Although in this study basal testosterone levels of subjects were low (approximately 3 ng/mL), after 14 and 28 days of supplementation, testosterone levels were not statistically significant and that is similar to the results obtained by Topo *et al* and Willoughby *et al* (40, 49). This is contrary with the hypothesis that D-Asp supplementation is effective with lower testosterone levels. 

A major limitation of the study was the small size of the sample groups. In addition, this study was open label and was not placebo controlled. However, no differences of statistical significance were observed between groups, hence it seems the allocation of participants in these groups have not been random. The latest study by Melville *et al* was a randomized, double-blinded, and placebo controlled design (51). Twenty four participants were randomly allocated to one of three experimental groups: placebo, 3 gr of D-Asp and 6 gr of D-Asp. 

Three gr of D-Asp acid had no significant effect on the testosterone levels after two weeks. It is similar finding by Bloomer *et al* and Willoughby *et al* and contrary to the study of Topo *et al* (40, 49, 50). Baseline testosterone levels of the current study were higher than values found in Topo *et al* and Bloomer *et al* experiments (6.3, 4.5 and 3 ng/mL respectively), so one can say that baseline testosterone levels may have no effect on influence of D-Asp supplementation that was previously assumed. In the six-gram group, total and free testosterone was significantly reduced from baseline. The authors hypothesized that 6 gr of D-Asp per day may affect negative feedback mechanisms of the HPG axis, thus reducing pituitary initiated production of luteinizing hormone and also testosterone levels. However, in the study of Bloomer *et al*, 6 gr daily of D-Asp had no effect on the testosterone levels (50). 

Probability this report revoked Melville *et al* hypothesis. The limitations of this research are the study length and small sample size. The observed reduction in testosterone may rebound, or even decrease further than expected.

**Table I:** Search strategy

**Search strategy to identify relevant exposures and outcomes:** 1. "D-ASP” or "d-aspartic acid" or "d-aspartate” 2. "Testosterone" or “LH” or "hypothalamic pituitary gonadal" or “HPG” or “Estradiol” or “SHBG” or “17 beta Hydroxy 4 Androsten 3 one”
**Search strings (all inclusive):** Parts 1 and 2 were combined using ‘AND’

**Table II:** The characteristics and outcomes of the included animal studies

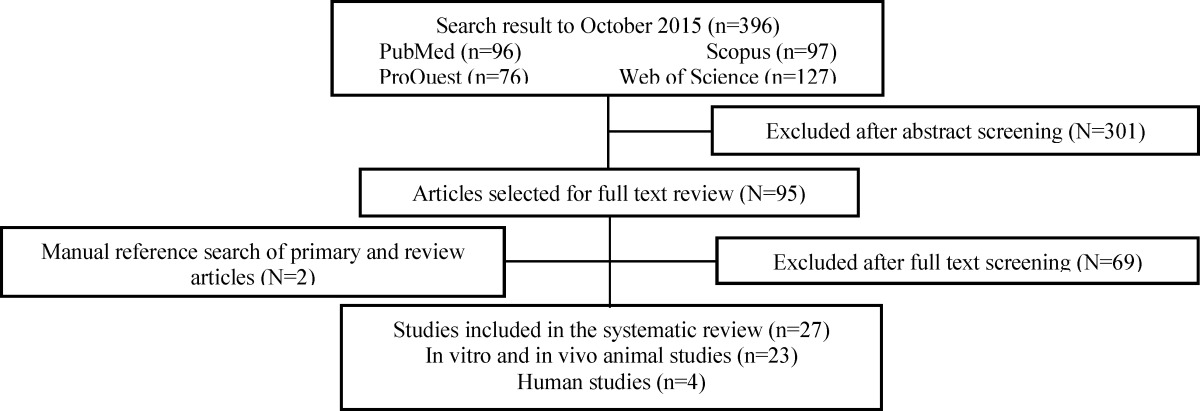

I.P.: Intraperitoneal injection min:

minute(s)

hr: hour(s)

wk: week(s)

CREB: cAMP response element binding protein

NMDAR: N-Methyl-D-Aspartic Acid receptor

StAR: steroidogenic acute regulatory protein

P450scc: cytochrome P450 cholesterol side-chain cleavage enzyme3b-

HSD: 3b-hydroxysteroid dehydrogenase/D5-D4 isomerases

**Table III:** The characteristics and outcomes of the included human studies

**First author, year (reference) ** **country**	**Design**	**Participants (Treatment Group)**	**Mean participants characteristics and age (year)**	**Exclusion criteria**	**Other participant demographics and comments**
Topo et al. 2009 (40) Italy	Placebo-controlled, parallel clinical trial	43 (23)	IVF (in vitro fertilization) patients (27-37)	No information given	Healthy sedentary
Willoughby et al. 2013 (49) USA	Randomized, double-blind, placebo-controlled, parallel clinical trial	20 (10)	Resistance trained men (resistance training for 1 year before the study) (22.8 ±4.67)	Consumption any nutritional supplements (excluding multivitamin) such as creatine monohydrate, nitric oxide-stimulating, hydroxy-β-methylbutyrate, or pharmacologic agents such as anabolic steroids 3 months before the study	Height, total body mass, all participants passed a mandatory medical screening
Bloomer et al. 2015 (50) USA	Parallel clinical trial	5 (One Serving) 5 (Two Servings)	Physically active men 40.8±1.0 (One Serving) 43.4±4.2 (Two Servings)	Smokers, history of cardiovascular or metabolic disease (including hypertension), using hormonal replacement or dietary supplements designed to increase hormone production	Age, height, body, weight, body mass index, waist circumference, hip circumference
Melville et al. 2015 (51) Australia	Randomize, double-blind, placebo-controlled, parallel clinical trial	24 (8) two groups	Resistance-trained men (minimum of two years’ experience in resistance training) (24.5 ± 3.2)	Acute or chronic medical conditions, consume any ergogenic or testosterone booting supplements	Training age, height, body mass, 1 RM bench

## Discussion

To our knowledge, this was the first systematic review to collect recent evidence on association between D-Asp and testosterone level. Although 396 articles identified from the search strategy, but only 27 articles; including 23 animal studies and 4 human studies satisfied search criteria. In vivo and in vitro animal studies revealed the mechanisms regulating D-Asp in the synthesis of steroid hormones with different effect of D-Asp depending on species, sex and organ-specific. 

The bulk of evidence revealed by these experiments suggests that the D-Asp in male animals alerts testosterone production in different ways by acting either directly on Leydig cells or indirectly on the hypothalamus-pituitary-testis axis (HPG axis):

1. D-Asp can directly increase the release of GnRH from the hypothalamus. Then GnRH induces the release of LH from the pituitary gland (14, 25)

2. D-Asp can be converted to NMDA that stimulates the release of GnRH in the hypothalamus (14).

3. D-Asp acts directly on the pituitary gland inducing an increase of LH releasing (14, 40).

4. D-Asp is capable of boosting the action of human chorionic gonadotropin (hCG), thereby it is able to induce testosterone synthesis in the Leydig cells (34, 46).

5. D-Asp increases the mRNA and protein levels of StAR (31, 45, 46).

6. D-Asp inhances aromatase activity, with consequent production of estradiol from testosterone (6, 42, 43).

7. D-Asp affects the gene expression of 5α-reductase, which converts testosterone into 5α-dihydrotestosterone (DHT) (31).

8. D-Asp participates in steroidogenesis through increasing the expression of some subunits of NMDA receptor in testes (44).

9. D-Asp administration induces an increase of c-kit receptor expression and of tyrosine kinase activity (41).

10. D-Asp induces up-regulation of androgen receptor and down-regulation of estrogen receptor expression (44).

11. D-Asp may delay LH receptor membrane trafficking mediated by hCG, resulting in an increased testosterone production (46).

Taken together, these data strongly support a prominent role of D-Asp in the neuroendocrine control of steroidogenesis activity. Therefore, the investigators assumed that this amino acid can be used as a testosterone booster for infertile men, and by athletes to increase muscle mass and strength. In our review, we were able to identify four studies on D-Asp supplementation have been conducted on humans that their results are contrary. The age, training status and basal testosterone values of subjects may be responsible for the difference in outcome between these studies.

The reviewed clinical trial studies included several design limitations by the following characteristics: short-term supplement duration studies (12-28 days), small sample sizes (N=10-48), lack of follow-up, no assessment the serum levels of D-Asp (except one), self-report for dietary intake and supplement compliance, limited descriptive information on the participants and a lack of moderator analysis. The small sample sizes most likely precluded the examination of important moderator variables such as age, weight status and basal testosterone levels. 

Although we show here that D-Asp has an important roles in synthesis and/or secretion of testosterone, high concentrations of D-Asp can have negative effects on some organs. Burrone *et al* showed that D-Asp increases the expression of superoxide dismutase 1 in the kidney and enhances caspase 3 levels in brain and heart tissues (53). These data indicate that exogenous D-Asp may induce oxidant stress and apoptosis in several tissues. Among included studies in this review only one study reported adverse effects of exogenous D-Asp (49). In human studies, doses of D-Asp were used ranging from 3 mg/d to 6 mg/d, which it is not clear that these doses are safe for humans or not. Additional data are not only required regarding the actual association of D-Asp with testosterone levels but also concerning the safe and maximum effective dose of D-Asp supplements for humans.

This study has several strengths. Frist, the comprehensive search was conducted on multiple databases and hand searching reference lists. Second, it may be the first systematic review focused on the relationship between D-Asp and testosterone concentration. Third, we included all type studies, which could more clearly explain this relationship. 

Some limitations also need to be considered. Frist, we only included English and published studies. Second, it is possible that we have missed some published studies. Finally, we do not enable to conduct meta-analysis due to high heterogeneity of the included studies. 

## Conclusion

This comprehensive systematic review showed that exogenous D-Asp enhances testosterone levels in male animal studies, whereas studies in human yielded inconsistent results. The evidence for this association in man is still sparse, mostly because of the lack of good quality studies. Based on limited and contrary of the data in humans, the paucity and often low quality of primary studies, it is clear that more and high-quality randomized controlled trials need to be conducted on D-Asp supplementation regarding their ability to increase endogenous levels of testosterone and elucidating the mechanisms of its action in human research. 

## Conflict of interest

The authors declare that there are no conflicts of interest.
